# The Effect of Selected Exercise Programs on the Quality of Life in Women with Grade 1 Stress Urinary Incontinence and Its Relationship with Various Body Mass Indices: A Randomized Trial

**DOI:** 10.1155/2020/1205281

**Published:** 2020-07-02

**Authors:** Magdalena Ptak, Sylwester Ciećwież, Agnieszka Brodowska, Aleksandra Szylińska, Andrzej Starczewski, Iwona Rotter

**Affiliations:** ^1^Department of Medical Rehabilitation and Clinical Physiotherapy, Pomeranian Medical University in Szczecin, Poland; ^2^Department of Gynecology, Endocrinology and Gynecologic Oncology, Pomeranian Medical University in Szczecin, Poland

## Abstract

**Aims:**

The aim of the study was to analyze the influence gymnastics has on the quality of life (QOL) in women with grade 1 stress urinary incontinence (SUI) and to determine the relationship between the outcome and selected body weight indices: body mass index (BMI) and waist-to-hip ratio (WHR).

**Methods:**

A randomized study of 140 women (45-60 years) with grade 1 SUI. The subjects were randomly assigned to a 3-month training for pelvic floor muscles and a transverse abdominal muscle (PFM + TrA, *n* = 70) or PFM alone (*n* = 70). The QOL was determined with the questionnaire International Consultation on Incontinence Modular Questionnaire–Lower Urinary Tract Symptoms Quality of Life (ICIQ LUTS QOL), before and after the program.

**Results:**

Women with BMI < 30 kg/m^2^ benefited more from the PFM + TrA program with respect to physical limitations and embarrassment domains, whereas patients with a gynoid body type (WHR < 0.8) benefited more in terms of physical and social limitations, SUI-evoked emotions, severity measures, and embarrassment domains.

**Conclusions:**

After the PFM + TrA training, women with WHR < 0.8 had a better QOL than those with WHR > 0.8.

## 1. Introduction

Overweight and obesity predispose to stress urinary incontinence (SUI) in perimenopausal women. The risk of SUI is particularly high in women whose body mass index (BMI) exceeds 30 kg/m^2^ [1]. The reduction of body weight in patients with obesity or overweight results in lower incidence of SUI, lesser frequency of incontinence episodes, and a better quality of life (QOL) [2]. Waist-to-hip ratio (WHR) may be used to monitor the effect abdominal obesity has on the occurrence of SUI. WHR is considered an indicator of fat distribution in a body. A high WHR means that women's distribution of fat is in the intra-abdominal region of the body. Intra-abdominal pressure (IAP) occurs in such activities as coughing, sneezing, pushing, and either performing heavy physical work or practicing certain sports. An increase in IAP was proved to correlate significantly with pelvic floor load, and thus, to be likely to predispose to SUI [3, 4]. The first thing specialists usually recommend is a conservative treatment, namely, a PFM training. Other crucial elements include educating patients on proper behavior during the whole day in order not to increase IAP and supporting the mechanism of the urethra closure in case of IAP increase by activating the Knack maneuver (a rapid, strong, short PFM contraction that precedes the increase of IAP). A similar precontraction occurs naturally in women who are continent. When SUI is present, such a PFM contraction, preceding the increase of IAP, stabilizes the urethra and prevents leakage of urine [5]. In the study conducted by Townsend et al. [6] in a group of older women (54-79 years), BMI increment was associated with higher incidence of urge and mixed UI, whereas a larger waist circumference correlated with a more frequent occurrence of SUI. The study confirmed the connection between PFM and TrA, which was described by Sapsford et al. [7].

The aim of this study was to analyze the effects of various pelvic floor muscle exercise programs on a QOL in women with grade 1 SUI and to determine the relationship between the outcome and selected body weight indices: BMI and WHR.

## 2. Materials and Methods

### 2.1. Study Design

The protocol of the study was approved by the Local Bioethics Committee at the Pomeranian Medical University (decision no. KB0012/142/13 of 30.09.2013).

### 2.2. Settings

Women referred to the Department of Gynecology, Endocrinology and Gynecologic Oncology, Pomeranian Medical University in Police (Poland; previously, till 2016, Department of Genecology and Urogynecology) in 2013-2016 and were diagnosed with grade 1 SUI.

### 2.3. Participants

A total of 600 women were diagnosed with grade 1 SUI based on their medical histories and the *Ingelman*-*Sundberg scale* [8]—a survey based on the Gaudenz Incontinence Questionnaire and urodynamic testing with a Libra apparatus (Medical Measurement System B.V. MMS, Netherlands). In the Ingelman-Sundberg scale, the incontinence severity is graded according to the circumstances or physical activities provoking urinary leakage. Grade I is when UI occurs while coughing and sneezing; grade II, while running and picking up heavy objects; and grade III, while walking or climbing stairs. The inclusion criteria of the study were age 45–60, grade 1 SUI confirmed with *a cough test in a urodynamic study and in a gynecological examination*, lack of urge incontinence, lack of any genitourinary surgeries or other illnesses (e.g., hypertension, diabetes), *lack of estrogen*-*dependent neoplasm or breast cancer and lack of pelvic organ prolapse* (*stage 0 in Pelvic Organ Prolapse Quantification*) *in a gynecological examination* in medical histories, and a written informed consent to participate in the study. Women younger than 45 and older than 60, with grades of SUI other than grade 1, with pelvic organ prolapse (*higher than stage 0 in Pelvic Organ Prolapse Quantification*), *with estrogen-dependent neoplasm and breast cancer*, after genitourinary surgeries, or those who had been prescribed any kind of medicine permanently, were excluded from the study, along with the patients who had not expressed their written informed consent to participate. Eventually, the study included 150 women who fulfilled the abovementioned criteria. The baseline demographic characteristics of the study subjects—age and menopausal status and parity—were obtained, and their anthropometric parameters—body weight and body height—were determined with medical scales (TANITA SC-240 MA, Middlesex, UK) and a stadiometer (SECA-217, Seca GMBH&CO, Hamburg, Germany), respectively.

### 2.4. Primary Outcome

All patients completed a survey with a Polish version of the International Consultation on Incontinence Modular Questionnaire–Lower Urinary Tract Symptoms–Quality of Life (ICIQ LUTS QOL) [8, 9]. The ICIQ LUTS QOL included questions referring to the influence of SUI on the QOL in various domains: physical limitations (daily activities, physical activity, and travelling), SUI-evoked emotions (embarrassment, odor issues), and changes in personal relationships (with a partner, close friends, etc.). The survey consisted of 19 questions, each scored on a 4-item scale, from 1 to 4, where “1” meant nothing at all, “2” little, “3” moderately, and “4” very much. Hence, the overall score could have ranged from 19 to 76. The raw scores were transformed according to Hebbar [9, 10] based on the King's Health Questionnaire—a slightly older, extensive questionnaire for a QOL research. The reliability of the questionnaire, verified on the base of its internal consistency rate expressed by Cronbach's alpha, was high (0.717 and 0.844 prior to and after completing the exercise program, respectively).

All study subjects were prescribed vaginal estrogens (estriol suppositories, 0.5 mg, twice a week) and randomly assigned to one of the exercise groups: A, intervention (*n* = 75) or B, control (*n* = 75). The Modified Oxford Scale was applied to assess a PFM function (vaginal palpation), in the supine position. The contraction at levels 2 (“week contraction”) -5 (“strong contraction”) was considered to be correct. The training program for group A included pelvic floor muscle (PFM) exercises with a cocontraction of the transverse abdominal muscle (TrA), performed four times per week for a period of three months. Each session included three series of PFM exercises with 10 repetitions, with 60-70% of a maximal voluntary contraction (MVC) lasting for 6-8 seconds, followed by two series with 10 repetitions, with 30-60% of a MVC lasting for 1-2 seconds. The patients were asked to contract their PFMs while breathing out and to perform the Knack maneuver whenever they felt an urge to cough, sneeze, or laugh. The patients practiced together, in groups, under the direction of a qualified physiotherapist. The training program for group B was essentially the same, however, without the cocontraction of the TrA.

### 2.5. Secondary Outcome

**Figure 1:**
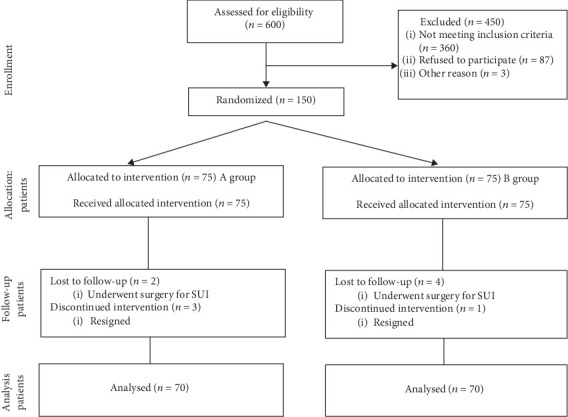
CONSORT diagram of the study [11]

After three months, the study subjects were surveyed with the ICIQ LUTS QOL again. Five patients from group A underwent a transobturator tape (TOT) procedure and hence did not complete the exercise program, and five women from group B were lost to follow-up. Eventually, the study included the results of 140 patients, 70 from group A and 70 from group B. CONSORT diagram of the study is presented in Figure 1.

### 2.6. Statistical Methods

The statistical characteristics of quantitative variables were presented as arithmetic means, standard deviations, medians, and minimum and maximum values. The results for qualitative variables were presented as numbers and percentages. Normal distribution of continuous variables was verified with the Shapiro-Wilk test. Statistical significance of differences between the study groups was verified with the Student *t*-test and Pearson chi-square test. The influence of BMI and WHR on the QOL was analyzed with the post hoc Tukey test for groups A and B, and BMI (BMI ≥ 30 kg/m^2^ vs. BMI < 30 kg/m^2^) and WHR (WHR ≥ 0.8 vs. WHR < 0.8). All calculations were carried out with Statistica 12 package (StatSoft, USA), with the threshold of statistical significance set at *p* < .05.

## 3. Results

The baseline characteristics of the study groups are presented in Table 1.

Women from groups A and B did not differ significantly in terms of their age, BMI, WHR, place of residence, declared levels of physical activity, menopausal status, and cigarette smoking status. The results of the post hoc Tukey, determining the effect of BMI (group 0 with BMI ≥ 30 kg/m^2^ vs. group 1 with BMI < 30 kg/m^2^) on the QOL scores at the end of two exercise programs, the PFM + TrA (group A) and the PFM only (group B), are shown in Table 2.

The post hoc analysis demonstrated a significant influence of BMI on the outcomes of the exercise training, expressed as physical limitations (Q4a) and embarrassment (QW) scores; beneficial effects of the PFM + TrA program on the QOL in these two domains were observed primarily in women with normal BMI (group A1). Irrespectively of their BMI, women participating in the PFM + TrA exercise program showed significantly better scores for social limitations (Q4b), SUI-evoked emotions (Q6), and severity measures (Q8), as well as significantly better overall QOL scores. However, social limitations (Q4b) scores in patients with excess body weight who participated in the PFM + TrA exercise program (group A0) were significantly better than in normal weight women assigned to the same training program (group A1). Neither BMI nor the training program affected significantly role limitations (Q3), social limitations (Q4b), relationship limitations (Q5), and sleep/energy (Q7) scores. The influence of the WHR (group 0 with an android body type, i.e., WHR ≥ 0.8 vs. group 1 with a gynoid body type, i.e. WHR<0.8) on the QOL scores at the end of the two exercise programs, the PFM + TrA (A) and the PFM (B), are presented in Table 3.

Irrespectively of their body type, women participating in the PFM + TrA exercise program showed significantly better overall QOL scores than those performing solely the PFM. However, beneficial effects of the PFM + TrA program on the QOL in five specific domains: physical limitations (Q4a), social limitations (Q4b), SUI-evoked emotions (Q6), severity measures (Q8), and embarrassment (QW) were observed primarily in patients with a gynoid body type (group A1).

## 4. Discussion

The researchers analyzing a link between obesity and SUI indicated an increase in IAP, which may lead to denervation of the pelvic floor. Moreover, body weight increment may also contribute to an increase in bladder pressure and to urethral hypermobility. Many authors pointed to the distribution of body fat as a significant determinant of a SUI risk. This implies that not only BMI but also WHR should be considered during the risk assessment. Obesity is known to predispose to urinary incontinence, especially SUI [12]. A decrease in BMI is associated with a better QOL and a lower number of incontinence episodes [13]. Furthermore, a relationship between WHR and SUI was highlighted by some authors [14], who suggested that while BMI correlates with the occurrence of mixed UI and the number of incontinence episodes, the incidence of SUI seems to be WHR dependent. Indeed, Subak et al. [15] showed that a reduction of body weight with a concomitant decrease in waist circumference by at least 3% resulted in up to 50% lower incidence of UI.

In this study, we analyzed the influence of BMI and WHR on the outcomes of conservative treatment of SUI, expressed as QOL scores. The survey with the ICIQ LUTS QOL showed that BMI contributed to statistically significant intergroup differences in physical limitations (Q4a) and embarrassment (QW) scores. Women with normal BMI (group A1), declared a significant improvement in these two QOL domains after the PFM + TrA exercise program. According to Han et al. [14], patients consider their ability to travel and undertake uninterrupted physical activities as more important domains of a QOL than their social life. Fitz et al. [16] confirmed that SUI-imposed limitations in physical activities were significantly reduced after implementation of (only) PFM exercises. Mean BMI of women participating in Fitz et al.'s study was 28.9 ± 5.2 kg/m^2^ as compared with 27.4 ± 4.6 kg/m^2^ in our study subjects. Although embarrassment is a common problem in patients with SUI, this issue has not been studied adequately thus far. Nevertheless, there is no doubt that this factor is a key determinant of a QOL. This hypothesis was supported by the results published by Elenskaia et al. [17] who showed that women considered the incontinence episodes more embarrassing than depression or cancer. Our patients who participated in the PFM + TrA exercise program (A) reported less social limitations (Q4b). Importantly, a more evident improvement in this QOL domain was observed in patients with higher baseline body weight (BMI 0). Perhaps this finding may be explained by a beneficial effect of the training program, which included not only exercises for the PFM but also for the TrA, a synergistic muscle involved in postural control [18]. Social limitations in the ICIQ LUTS QOL refer to interpersonal contacts, ability to see/visit friends and family life. Perhaps the TrA exercises contributed to better postural control in the study subjects, and the PFM training enabled them to gain control over their PFM. Thus, despite the excess body weight, they were more confident in their relationships with others, which might contribute to the higher overall QOL scores. Body posture, in particular anterior pelvic tilt, may play a role in the development of UI. Whenever pelvic organs slip down forward, the load of urogenital diaphragm increases considerably. Changes in body posture and their beneficial effects on pelvic floor were studied by Capson et al. [19]. The number of statistically significant intergroup differences in the QOL scores increased considerably when WHR was considered as a covariate. The results of the survey with the ICIQ LUTS QOL suggest that women with a gynoid body type benefited more from the PFM + TrA program, demonstrating less physical (Q4a) and social limitations (Q4b), less SUI-evoked emotions (Q6) and lesser embarrassment (QW), and undertaking less often various activities included in Q8 domain, such as wearing pads, controlling the amount of ingested fluids, etc. A perimenopausal period is usually associated with an increase in WHR, which results in lower concentration of growth hormone, decrease in a sympathetic activity and redistribution of adipose tissue [20]. In the classification of risk factors for UI proposed by Bump, obesity is listed among decompensating medical conditions. While the link between BMI and UI is an established phenomenon, the incidence of this condition may also depend on WHR. Analyzing the outcomes of a hormone replacement therapy in postmenopausal women, Brown et al. [21] demonstrated that patients with higher WHR more often showed SUI, but not other types of urinary incontinence. This relationship was also confirmed by other authors who showed that the number of SUI episodes increased with WHR [22]. Finally, Subak et al. [15] demonstrated that in women subjected to weight reduction intervention, WHR was a more accurate marker of changes in incontinence frequency than BMI.

## 5. Conclusion

Participation in the PFM + TrA exercise program exerted a beneficial effect on the QOL in the patients with grade 1 SUI. While BMI had a limited effect on the outcomes of the PFM + TrA training, the patients with a gynoid body type (WHR < 0.8) benefited more in terms of the QOL than women with an android body type (*WHR* ≥ 0.8).

### 5.1. Limitations

The authors point to the fact that the study was carried out in a group of 140 patients. In reference to the large numbers of SUI occurrences at various stages of a woman's life, it is important to expand the number of analyzed patients in the future and to attempt to increase the age range in order to achieve reliable results.

## Figures and Tables

**Table 1:** The baseline characteristics of the study patients randomly assigned to the PFM + TrA (group A) and PFM training (group B).

Parameter	Category	Group A (*n* = 70)	Group B (*n* = 70)	*p* value
Age (years)		53.1 ± 5.5	53.0 ± 5.7	0.813^∗^
BMI (%)	Group 0 ≥ 30 kg/m^2^	26.0	25.0	0.871
	Group 1 < 30 kg/m^2^	74.0	75.0	
WHR (%)	Group 0 ≥ 0.8	21.4	50.0	0.088
	Group 1 < 0.8	78.6	50.0	
Place of residence (%)	City/town	75.7	77.1	0.842
	Countryside	24.3	22.9	
Physical activity level (%)	Sedentary	12.9	18.6	0.616
	Active	30.0	25.7	
	Mixed	57.1	55.7	
Menopausal status (%)	Premenopausal	47.1	61.4	0.090
	Postmenopausal	52.9	38.6	
Smoking status (%)	Smokers	12.9	11.4	0.800
	Nonsmokers	87.1	88.6	
Number of natural deliveries	≥3	4	9	0.301
	<3	96	91	

BMI: body mass index; WHR: waist-to-hip ratio; *x*˜:average; SD: standard deviation. ^∗^Student *t*-test test, *p*: Pearson chi^2^ test.

**Table 2:** The influence of BMI on the QOL after the exercise training**—**results of the post hoc Tukey test.

Variable	Exercise group	BMI group	Score	*p* value (post hoc test)
Role limitations (Q3)	A	0	16.3 ± 16.9	A0 vs. B0 *p* = 0.080
		1	23.1 ± 26.9	A1 vs. B1 *p* = 0.375
	B	0	27.9 ± 18.3	A0 vs. A1 *p* = 0.929
		1	37.9 ± 23.4	B0 vs. B1 *p* = 0.625
Physical limitations (Q4a)	A	0	18.9 ± 14.8	A0 vs. B0 *p* = 0.130
		1	18.5 ± 16.1	A1 vs. B1 *p* = 0.019
	B	0	30.8 ± 22.2	A0 vs. A1 *p* = 0.999
		1	41.7 ± 16.4	B0 vs. B1 *p* = 0.537
Social limitations (Q4b)	A	0	5.9 ± 8.9	A0 vs. B0 *p* < 0.001
		1	9.6 ± 11.6	A1 vs. B1 *p* = 0.003
	B	0	19.3 ± 15.7	A0 vs. A1 *p* < 0.001
		1	30.2 ± 16.9	B0 vs. B1 *p* = 0.218
Relationship limitations (Q5)	A	0	15.1 ± 26.9	A0 vs. B0 *p* = 0.519
		1	14.8 ± 18.4	A1 vs. B1 *p* = 0.717
	B	0	25.3 ± 28.9	A0 vs. A1 *p* = 0.999
		1	29.6 ± 18.6	B0 vs. B1 *p* = 0.997
Emotions (Q6)	A	0	10.7 ± 13.0	A0 vs. B0 *p* < 0.003
		1	13.6 ± 17.3	A1 vs. B1 *p* = 0.004
	B	0	24.8 ± 19.4	B0 vs. B1 *p* = 0.997
		1	37.0 ± 22.5	B0 vs. B1 *p* = 0.237
Sleep/energy (Q7)	A	0	19.6 ± 18.9	A0 vs. B0 *p* = 0.058
		1	20.4 ± 23.3	A1 vs. B1 *p* = 0.136
	B	0	32.4 ± 19.6	A0 vs. A1 *p* = 0.624
		1	39.8 ± 16.3	B0 vs. B1 *p* = 0.997
Severity measures (Q8)	A	0	23.9 ± 16.6	A0 vs. B0 *p* < 0.001
		1	25.5 ± 21.9	A1 vs. B1 *p* = 0.004
	B	0	40.5 ± 17.6	A0 vs. A1 *p* = 0.999
		1	50.0 ± 14.6	B0 vs. B1 *p* = 0.647
Embarrassment (QW)	A	0	14.1 ± 21.2	A0 vs. B0 *p* = 0.237
		1	16.7 ± 20.6	A1 vs. B1 *p* = 0.044
	B	0	26.9 ± 24.7	A0 vs. A1 *p* = 0.999
		1	44.4 ± 28.0	B0 vs. B1 *p* = 0.261
Overall QOL	A	0	111.4 ± 75.9	A0 vs. B0 *p* < 0.001
		1	125.2 ± 111.7	A1 vs. B1 *p* < .001
	B	0	200.9 ± 89.9	A0 vs. A1 *p* = 0.999
		1	266.4 ± 77.1	B0 vs. B1 *p* = 0.144

BMI group 0 = BMI > 30 kg/m^2^; BMI group 1 = BMI ≤ 30 kg/m^2^; group A (BMI group 0 *n* = 50; BMI group 1 *n* = 20); group B (BMI group 0 *n* = 52; BMI group 1 *n* = 18); $\overset{®}{x}$: average; SEM: standard error of the mean; A0: scores in the A group and BMI > 30 kg/m^2^; A1: scores in the A group and BMI ≤ 30 kg/m^2^; B0: scores in the B group and BMI > 30 kg/m^2^; B1: scores in the B group and BMI ≤ 30 kg/m^2^.

**Table 3:** The influence of WHR on the QOL after the exercise training—the results of the post hoc Tukey test.

Variable	Exercise	WHR	Score	*p* value (post-hoc test)
	Group	Group		
Role limitations (Q3)	A	0	22.2 ± 14.9	A0 vs. B0 *p* = 0.904
		1	16.9 ± 21.2	A1 vs. B1 *p* = 0.054
	B	0	30.5 ± 15.4	A0 vs. A1 *p* = 0.989
		1	30.5 ± 24.0	B0 vs. B1 *p* = 0.999
Physical limitations (Q4a)	A	0	26.7 ± 16.4	A0 vs. B0 *p* = 0.877
		1	17.6 ± 14.1	A1 vs. B1 *p* = 0.027
	B	0	35.2 ± 18.4	A0 vs. A1 *p* = 0.999
		1	31.9 ± 24.0	B0 vs. B1 *p* = 0.997
Social limitations (Q4b)	A	0	8.9 ± 10.5	A0 vs. B0 *p* = 0.092
		1	6.3 ± 9.5	A1 vs. B1 *p* = 0.002
	B	0	23.5 ± 18.0	A0 vs. A1 *p* = 0.999
		1	20.6 ± 15.3	B0 vs. B1 *p* = 0.997
Relationship limitations (Q5)	A	0	12.2 ± 19.4	A0 vs. B0 *p* = 0.501
		1	15.8 ± 26.3	A1 vs. B1 *p* = 0.825
	B	0	28.6 ± 28.2	A0 vs. A1 *p* = 0.999
		1	24.2 ± 25.0	B0 vs. B1 *p* = 0.997
Emotions (Q6)	A	0	11.9 ± 11.5	A0 vs. B0 *p* = 0.085
		1	11.3 ± 14.9	A1 vs. B1 *p* = 0.001
	B	0	28.3 ± 20.8	A0 vs. A1 *p* = 0.999
		1	27.6 ± 21.1	B0 vs. B1 *p* = 0.998
Sleep/energy (Q7)	A	0	22.2 ± 22.4	A0 vs. B0 *p*= 0.396
		1	19.1 ± 19.4	A1 vs. B1 *p* = 0.124
	B	0	36.7 ± 19.7	A0 vs. A1 *p* = 0.624
		1	31.9 ± 18.2	B0 vs. B1 *p* = 0.986
Severity measures (Q8)	A	0	32.8 ± 18.2	A0 vs. B0 *p* = 0.366
		1	21.9 ± 17.3	A1 vs. B1 *p* < 0.001
	B	0	45.7 ± 19.1	A0 vs. A1 *p* = 0.533
		1	40.2 ± 15.0	B0 vs. B1 *p* = 0.935
Embarrassment (QW)	A	0	20.0 ± 30.3	A0 vs. B0 *p* = 0.974
		1	13.3 ± 17.7	A1 vs. B1 *p* = 0.010
	B	0	28.6 ± 23.1	A0 vs. A1 *p* = 0.991
		1	34.3 ± 29.7	B0 vs. B1 *p* = 0.989
Overall QOL	A	0	136.9 ± 82.4	A0 vs. B0 *p* = 0.030
		1	108.9 ± 86.6	A1 vs. B1 *p* < 0.001
	B	0	228.4 ± 83.4	A0 vs. A1 *p* = 0.967
		1	207.1 ± 97.9	B0 vs. B1 *p* = 0.978

WHR group 0 = WHR ≥ 0.8; WHR group 1 = WHR < 0.8; group A (WHR group 0 *n* = 15; WHR group 1 *n* = 55); group B (WHR group 0 *n* = 35; WHR group 1 *n* = 35); $\overset{®}{x}$: average; SEM: standard error of the mean; A0: scores in A group and WHR ≥ 0.8; A1: scores in A group and WHR < 0.8; B0: scores in B group and WHR ≥ 0.8; B1: scores in B group and WHR < 0.8.

## Data Availability

The data used to support the findings of this study are available from the corresponding author upon request.
